# A validated composite organ and hematologic response model for early assessment of treatment outcomes in light chain amyloidosis

**DOI:** 10.1038/s41408-020-0306-5

**Published:** 2020-04-14

**Authors:** Surbhi Sidana, Paolo Milani, Moritz Binder, Marco Basset, Nidhi Tandon, Andrea Foli, Angela Dispenzieri, Morie A. Gertz, Suzanne R. Hayman, Francis K. Buadi, Martha Q. Lacy, Prashant Kapoor, Nelson Leung, S. Vincent Rajkumar, Giampaolo Merlini, Giovanni Palladini, Shaji K. Kumar

**Affiliations:** 1 Division of Hematology, Department of Internal Medicine, Mayo Clinic, Rochester, MN USA; 2 Department of Medicine, Stanford University, Stanford, CA USA; 3 Amyloidosis Research and Treatment Center, Foundation “Istituto di Ricovero e Cura a Carattere Scientifico (IRCCS) Policlinico San Matteo”, Pavia, Italy; 4 Department of Molecular Medicine, University of Pavia, Pavia, Italy

**Keywords:** Risk factors, Haematological cancer

## Abstract

Newly diagnosed AL amyloidosis patients were evaluated to develop a model for early assessment of treatment benefit at 6 months, integrating both hematologic (HR) and organ response (OR) assessment (testing cohort, Mayo: *n* = 473; validation cohort, Pavia: *n* = 575). Multiple OR were assessed as follows: All OR (AOR): response in all organs, mixed OR (MOR): response in some organs, no OR (NOR)]. AOR rates at 6 months improved with deepening HR; complete response (CR; 38%, 35%), very good partial response (VGPR; 30%, 26%), and partial response (PR; 16%, 21%), respectively. A composite HR/OR (CHOR) model was developed using incremental scoring based on hazard ratios with scores of 0–3 for HR (0—CR, 1—VGPR, 2—PR, 3—no response) and 0–2 for OR (0—AOR, 1—MOR, 2—NOR). Patients could be divided into two distinct CHOR groups (scores 0–3 and 4–5), with median OS in group 1 and group 2: Not reached vs. 34 months, *p* < 0.001 [Mayo] and 87 vs. 23 months, *p* < 0.001 [Pavia]. In conclusion, we developed a model that can assess multiple organs concurrently, and integrate both HR and OR assessments to determine early clinical benefit with treatment, which may be used as a surrogate end-point in trials and to compare outcomes with different therapies.

## Introduction

Deposition of misfolded light chains secreted by the plasma cell clone leads to organ dysfunction in patients with light chain (AL) amyloidosis^1–3^. The most commonly affected organs include the heart, kidney, and liver; and many patients have more than one organ involvement^3–5^. Prognosis depends both on the severity of organ involvement, especially the heart, and the underlying plasma cell burden^6–8^. Treatment is targeted toward the plasma cell clone^2,9–13^. In most patients, the organ dysfunction is the main driver of morbidity and mortality and the plasma cell burden is usually low^6,8,14,15^. Given this, it would be ideal to assess treatment efficacy by its impact on organ improvement. However, time to organ response (OR) can be varied and is usually delayed^5^. Therefore, treatment efficacy, especially early-on is typically determined by hematologic response (HR)^16^.

Deep HR increases the likelihood of OR and long-term survival, but this is not always the case and there is inter-patient variability in the relationship between depths of HR and OR^5,17^. No model currently exists to integrate the two assessments for clinical use. This makes early assessment of treatment benefit difficult in this disease, preventing relatively rapid evaluation of clinical trial results and precludes design of clinical trials for timely intervention in patients with likely poor outcome with ongoing therapies. Early identification of patients who are not likely to benefit from a given therapy is increasingly important as these patients have an inferior survival and more treatment options are becoming available for these patients^3^. A composite model that takes into account both HR and OR at a given time point may allow for early assessment and thus become a useful surrogate endpoint for clinical trials. It may also identify patients who have not achieved a deep HR, but can safely continue first line therapy if they have achieved an OR. Such a surrogate model may also be helpful in light of new, emerging therapies that target the amyloid fibril and can potentially lead to earlier OR^18,19^.

In this study, we have developed and validated a composite model to integrate OR and HR in AL amyloidosis to define a surrogate end point for use in treatment trials in AL amyloidosis.

## Patients and methods

### Study population

Patients with biopsy proven newly diagnosed AL amyloidosis with involvement of heart, liver, or kidney who received treatment were included. Amyloid deposits were confirmed as AL type by electron microscopy immunohistochemistry^20^ or mass spectrometry^21^. We identified 875 patients diagnosed from 1/1/2006 to 12/31/2015 from the Mayo Clinic dataset, of which 473 patients had HR and OR data available at 6 months (test cohort; Supplementary Fig. 1 consort flow diagram). The validation cohort included 575 patients from the Pavia Amyloidosis Research and Treatment Center dataset. Both datasets are maintained prospectively with approval by the respective Institutional Review Committees.

### Response assessment

In evaluable patients, HR was assessed using validated criteria^16^. Patients who had difference in involved and uninvolved free light chains (dFLC) <5 mg/dL were assessed for response by criteria of complete response (CR) only, based on recent data^22–24^. Organ involvement and response were assessed by using existing criteria as described in supplementary data^16,25–27^. OR was classified as all organ response (AOR): response in all of the involved and evaluable organs (heart, kidney, liver); mixed organ response (MOR): response in at least one of the organs and no organ response (NOR). Patients were assessed for response at the 6 months (±2 months) and 12 months (±2 months) time-point in the Mayo cohort and at 6 months time-point (±2 months) for the Pavia group.

### Combined hematologic and OR (CHOR) model

**Fig. 1:**
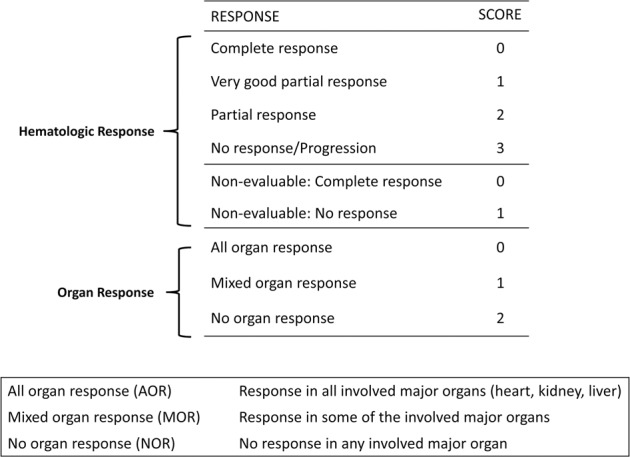
Scoring system for composite hematologic and organ response (CHOR) model.

A model for CHOR was developed using the Mayo Clinic test cohort (Fig. 1). Patients were assigned scores of 0–3 for HR as follows: 0—CR, 1—very good partial response (VGPR), 2—partial response (PR), 3—no response (NR) or progression. Patients who had dFLC <5 mg/dL were assigned a score of 0 for CR and 1 for other response as OS for the latter group was most similar to achieving VGPR. OR was scored as follows: 0—AOR, 1—MOR, and 2—NOR. Hazard ratios (HR) for OS were calculated for scores 1–5 relative to a score 0 (complete OR and HR) to construct groups based on similar hazard ratios. Patients were then divided into two groups: CHOR group 1 (scores 0–3) and CHOR group 2 (scores 4–5).

### Analysis

Statistical analysis was carried out using the JMP (version 12, SAS Institute Inc., Cary, NC) and Stata (version 13.1, StataCorp, College Station, TX) software for the Mayo Clinic cohort and using MedCalc Statistical Software version 18.1 (MedCalc Software bvba, Ostend, Belgium; http://www.medcalc.org) for the Pavia cohort. Chi-Square and Fischer Exact tests were used to carry out univariate analysis for categorical variables and Wilcoxon Rank Sum/Kruskal–Wallis for continuous variables. Survival analysis was carried out using the Kaplan–Meier method and the log-rank test was used to compare survival curves. Cox proportional hazards model was used to evaluate hazard ratios for survival. 95% confidence intervals (CI) are reported. OS was defined as the time from start of treatment to death. Cox regression was used to compare the predictive power of HR, OR, and the composite CHOR model^28^. Goodness of fit of nested models was evaluated using likelihood ratio tests. Predictive power of the individual and composite models were compared using Harrell’s C^29^. All hypothesis tests were two-sided, *p*-values below 0.05 were considered statistically significant.

## Results

### Baseline characteristics

The test cohort consisted of 473 patients from Mayo Clinic cohort, who were alive at the 6-month time point and had HR and OR data available. The validation cohort consisted of 575 patients from Pavia with response data available at the 6-month time-point. Baseline characteristics of patients in the two cohorts are summarized in Table 1. The median age at diagnosis was 63–64 years in both series, and males comprised 65% and 58% of the population in the Mayo and Pavia cohorts, respectively. Amyloidogenic light chain was lambda in 78% of patients in both cohorts. Median dFLC and bone marrow plasma cells at diagnosis were: 19 mg/dL and 10% (Mayo) and 19 mg/dL and 11% (Pavia), respectively. Proportion of patients with dFLC < 5 mg/dL (or 50 mg/L) at diagnosis was 13% in the Mayo cohort and 7% in the Pavia cohort. Presence of t(11,14) on interphase fluorescence in-situ hybridization (iFISH) was noted in 53% of patients and presence of trisomy/tetrasomy in 21% of patients in the Mayo cohort. iFISH data of the Pavia cohort were not available. The most common organs involved were the heart (70% and 79%) and kidney (70% and 69%). Liver was involved in 14% and 11% of patients, respectively. Number of major organs (heart, liver, and kidney) involved in patients from the Mayo cohort were as follows, one: 54%, two: 37%, and three: 8%. In the Pavia cohort, distribution of organ involvement was as follows: one: 47%, two: 46%, and three: 7%.

**Table 1:** Baseline characteristics and organ involvement.

	Mayo cohort *N* = 473 *N* (%) or Median (IQR)	Pavia cohort, *N* = 575 *N* (%) or Median (IQR)
Median age, years	63 (56–69)	64 (56–70)
Sex, males	309 (65)	335 (58)
Involved light chain, lambda	354/467 (78)	450/575 (78)
Median M-protein (g/dL)	0 (0–0.6)	0.8 (0–1.5)
Median dFLC (mg/dL)	19 (8–55)	19 (9–52)
dFLC <5 mg/dL	63 (13)	39 (7)
Bone marrow plasma cells (%)	10 (5–12)	11 (7–20)
NT-Pro BNP (pg/mL)	1625 (327–4296)	2215 (704–5578)
Troponin-T (ng/mL)	0.02 (0.01–0.05)	Not available
Troponin-I (ng/mL)	Not available	0.05 (0.02–0.13)
Median 24 h urine protein (mg)	2629 (375–7008)	2561 (392–6590)
Median GFR (ml/min/1.73 m^2^ BSA)	65 (48–82)	69 (49–87)
Median alkaline phosphatase (U/L)	86 (69–118)	149 (88–217)
Mayo 2012 stage 1/2/3/4	125/123/115/93 (27/27/25/20) (*N* = 456)	109/159/151/111 (20/30/28/22) (*N* = 530)
Mayo 2004 stage 1/2/3a/3b	107/205/108/37 (23/45/24/8) (*N* = 457)	78/276/107/69 (15/52/20/13) (*N* = 530)
Renal stage 1/2/3 (%)	229/183/53 (49/39/11), *N* = 465	285/213/68 (50/37/13) *N* = 566
iFISH—t (11; 14)	172/325 (53)	Not available
iFISH—trisomy/tetrasomy	67/326 (21)	Not available
*Organ involvement*		
Heart	332 (70)	454 (79)
Kidney	330 (70)	399 (69)
Liver	66 (14)	64 (11)
Gastrointestinal system	101 (21)	13 (2)
Autonomic nervous system	47 (10)	61 (10)
*Major organ involved*^a^		
One major organ involved	255 (54)	269 (47)
Two major organs involved	181 (37)	268 (46)
Three major organs involved	37 (8)	38 (7)
More than one major organ involved	218 (46)	306 (53)

*BSA* body surface area, *dFLC* difference in involved and uninvolved free light chains, *GFR* Glomerular filtration rate, *IQR* interquartile, *iFISH* interphase fluorescence in situ hybridization, *M-protein* monoclonal protein.

^a^Major organs: heart, kidney, and liver.

### Treatment and Hematologic Response

First-line treatment received by the 473 patients in the Mayo cohort was as follows: autologous stem cell transplant (ASCT) with or without induction therapy, 41% (*n* = 194); bortezomib-based chemotherapy, 21% (*n* = 100); alkylator-based therapy, 35% (*n* = 165), immunomodulatory drugs 3% (*n* = 13), and steroids/other in one patient. HR rates at 6 months in the 410 evaluable patients were as follows: CR was seen in 25% (101/410), VGPR in 35% (144/410), PR in 24% (99/410), while 16% (66/410) of patients had NR/disease progression. There were 63 patients who were not evaluable given baseline dFLC <5 mg/dL. Of these, 29% (18/63) achieved a CR. In the Pavia cohort, treatment received was as follows: bortezomib-based chemotherapy, 44% (*n* = 255), melphalan and dexamethasone, 40% (*n* = 234), immunomodulatory drugs 9% (*n* = 52), ASCT, 1% (*n* = 9), and steroids/other 4% (*n* = 25). HR rates at 6 months were: CR: 14% (74/536), VGPR: 33% (176/536), PR: 19% (105/536), while 34% of patients had NR/disease progression (181/536). Remaining 39 patients had dFLC< 5 mg/dL and all achieved a CR.

#### Organ Response Rates

##### Individual OR

For individual OR assessment, patients on dialysis at diagnosis were excluded from renal response assessment (Mayo: 4%; *n* = 14; Pavia: 1%, *n* = 9). In the Mayo cohort, cardiac response was seen in 25% (73/293), renal response in 34% (103/300), and liver response in 25% (16/64) of patients evaluated at the 6 months time-point, respectively. Rates of OR at 6 months in the Pavia cohort were: cardiac: 27% (116/424), renal: 36% (135/375), and liver: 17% (9/52), respectively.

##### Combined OR

Combined OR in the Mayo Clinic cohort at 6 months was as follows: AOR 26% (125/473), MOR: 14% (*n* = 66/473), NOR: 60% (*n* = 282/473). OR rates improved at 12 months; AOR: 45% (*n* = 194/435), MOR: 12% (*n* = 54/435) and NOR: 43% (*n* = 187/435). Combined OR rates at 6 months in the Pavia cohort were as follows: AOR 21% (*n* = 120/575), MOR: 18% (*n* = 105/575), NOR: 61% (*n* = 350/575). Combined OR rates increased with deeper HR. In the Mayo cohort, OR rates at 6 months for patients achieving hematologic CR (including CR in dFLC < 5 mg/dL) were: AOR 38% (45/119), MOR: 13% (15/119), and NOR 50% (59/119). OR in the hematologic VGPR group (including dFLC < 5 mg/dL without CR) were AOR: 30% (57/189), MOR: 16% (31/189), and NOR: 53% (101/189) and in the hematologic PR group were AOR: 16% (16/99), MOR: 18% (18/99), and NOR 66% (65/99). In the Pavia cohort, OR rates for patients achieving hematologic CR were: AOR 35% (40/113), MOR: 23% (26/113), and NOR 41% (47/113). OR in the hematologic VGPR group were AOR: 26% (46/176), MOR: 22% (39/176), and NOR: 51% (91/176) and in the hematologic PR group were AOR: 21% (22/105), MOR: 20% (21/105), and NOR 59% (62/105).

In the Mayo Clinic group, there were nine patients who either had no HR or hematologic progression at 6 months, who had concurrent OR. There were three patients with both kidney and renal involvement; two of them had only renal response and one had both heart and renal response. The remaining six patients had renal involvement alone and had a renal response. In the Pavia cohort at 6 months there were seven patients who obtained a cardiac response and 24 patients who had a renal response, while no HR was achieved. However, in all cases a reduction of dFLC between 40% and 50% was achieved.

#### Survival

**Fig. 2:**
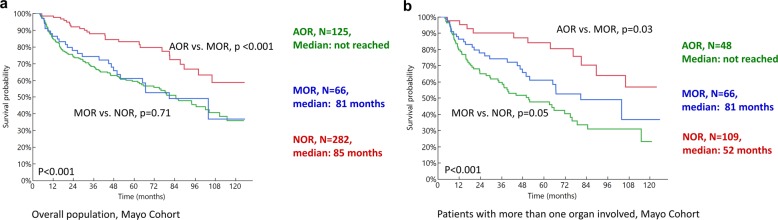
Overall survival in the Mayo Clinic cohort based on achieving all organ response (AOR), mixed organ response (MOR) and no organ response (NOR) at 6 months from start of first-line therapy. **a** All patients, **b** patients with more than one organ involved.

**Fig. 3:**
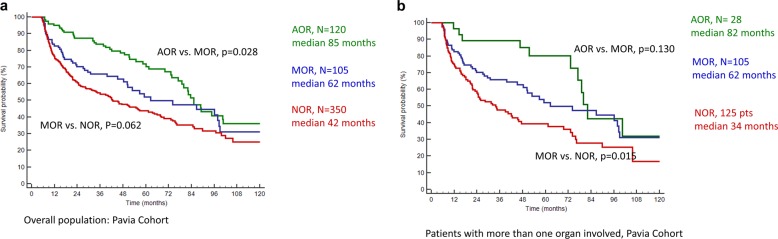
Overall survival in the Pavia cohort based on achieving all organ response (AOR), mixed organ response (MOR) and no organ response (NOR) at 6 months from start of first-line therapy. **a** All patients, **b** patients with more than one organ involved.

Figure 2 and Table 2 describe the survival outcomes in the Mayo cohort based on the combined OR parameter for the 6-month response (Fig. 2a, b). Survival based on combined OR was analyzed for all patients (Fig. 2a) and subsets of patients with more than one organ involved (Fig. 2b). Patients who achieved AOR at 6 months had the best outcomes with median OS in AOR vs. MOR vs. NOR groups being:, not reached vs. 81 vs. 85 months, *p* < 0.001. In patients with more than one organ involved, where patients could have mixed or discordant ORs, median OS in the three groups (AOR vs. MOR vs. NOR) was not reached. vs. 81 vs. 52 months. These parameters were evaluated in the Pavia cohort based on 6-month OR and were predictive of OS (Table 2, Fig. 3a, b). Patients with AOR had the best survival, followed by MOR and NOR (Fig. 3a). Subset analyses in patients with more than one organ involved showed similar results (Fig. 3b and Supplementary Fig. 2). While the absolute survival outcomes were different in the Mayo Clinic and Pavia cohort, the survival trend and magnitude of difference was similar across the two groups. In the subset of patients with heart involvement, OS based on AOR vs. MOR vs. NOR was as follows in the Mayo Cohort: not reached vs. 81 months vs. 63 months, *p* < 0.001 and similar in the Pavia cohort (Table 2 and Supplementary Fig. 3).

**Table 2:** Overall survival outcomes based on combined organ response and composite hematologic and organ response (CHOR) model.

	Mayo Clinic cohort				Pavia cohort			
	*N*	Median OS (95% CI)	*p*-value	Hazard ratio (95% CI), *p*-value	*N*	Median OS (95% CI)	*p*-value	Hazard ratio (95% CI), *p*-value
*Combined organ response*								
Overall population	473				575			
AOR	125	NR (98-NR)		Reference	120	85 (79–100)		Reference
MOR	66	81 (50-NR)	<0.001^a^	2.2 (1.3–3.8), *p* = 0.003	105	62 (50–98)	0.028^a^	1.6 (1.0–2.3), *p* = 0.029
NOR	282	85 months (66–105)	0.71^b^	2.4 (1.6–3.7), *p* < 0.001	350	42 (33–56)	0.062^b^	2.1 (1.5–2.0), *p* < 0.001
>1 organ involved	235				258			
AOR	48	NR (81-NR)		Reference	28	82 (77–100)		
MOR	66	81 months (50-NR)	0.03^a^	2.1 (1.1–4.0), *p* = 0.02	105	62 (50–98)	0.130^a^	1.6 (0.8–3.1), *p* = 0.13
NOR	109	52 months (35–75)	0.05^b^	2.9 (1.7–5.0), *p* < 0.001	125	34 (23–47)	0.015^b^	2.6 (1.4–5.0), *p* = 0.002
Heart involvement	332				424			
AOR	68	NR (81-NR)		Reference	72	84 (73–100)		Reference
MOR	63	81 months (50-NR)	0.05^a^	1.8 (1.0–3.3), *p* = 0.06	102	62 (50–98)	0.05^a^	1.4 (0.9-2-2), *p* = 0.11
NOR	201	63 months (40–78)	0.13^b^	2.5 (1.5–4.2), *p* < 0.001	250	25 (20–38)	0.001^b^	2.4 (1.6–3.5), *p* < 0.001
*CHOR model*	473				575			
Group-1	349	NR (103-NR)		Reference	344	87 (79–98)		Reference
Group-2	124	34 months (21–46)	<0.001	3.4 (2.5–4.6), *p* < 0.001	231	23 (18–30)	<0.001	2.8 (2.2–3.5), *p* < 0.001

*AOR* all organ response, *MOR* mixed organ response, *NOR* no organ response, *CHOR* composite hematologic and organ response, *NR* not reached, *OS* overall survival.

^a^Log-rank *p*-value for AOR vs. MOR.

^b^Log-rank *p*-value for MOR vs. NOR.

In patients with involvement of both heart and kidney and who achieved cardiac response by 6 months, status of renal response did not impact survival further as shown in *Supplementary data*. Amongst patients with renal involvement, achievement of renal response by 6 months was associated with significantly better dialysis-free survival, with 88% vs. 65% of patients remaining dialysis free at 5 years, *p* < 0.001 (Mayo cohort)

#### CHOR model

**Fig. 4:**
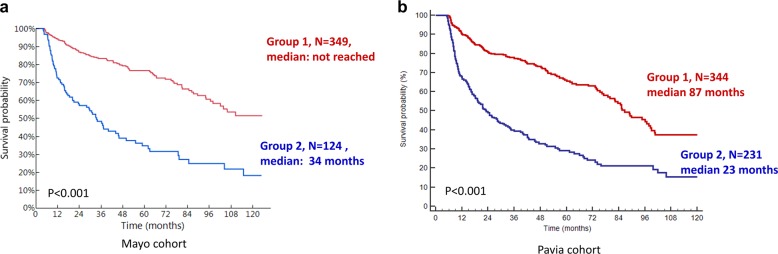
Overall survival by composite organ and hematologic response (CHOR) model. **a** Mayo Clinic cohort and **b** Pavia cohort.

A composite score (CHOR) was developed based on HR and OR as described in the “Methods” section (Fig. 1). Patients with a score of zero were those who achieved a hematologic CR as well as well as response in all organs. Patients were divided into two groups based on the HR for survival (Mayo cohort). The groups were as follows: group 1: scores of 0–3 (*N* = 349), group 2: scores of 4–5 (*N* = 124). As illustrated in Fig. 4a, b, patients in CHOR group 1 had significantly better survival outcome compared to group 2 (median OS: not reached vs. 34 months, *p* < 0.001) with HR of 3.4 (2.5–4.6), *p* < 0.001. This model was then validated in the Pavia cohort and median OS for patients in CHOR group 1 vs. 2 was 87 vs. 23 months, *p* < 0.001 with HR of 2.8 (2.2–3.5), *p* < 0.001. This model was also tested in sub-groups of patients with Mayo 2012 stages I–IV and at 12 months in the Mayo cohort with similar results (Supplementary Figs. 4 and 5).

We compared the CHOR model (group 1 vs. 2) at the 6-month time-point to (1) the HR criteria (achieving CR vs. not) and (2) achieving AOR vs. not using Cox regression, with Mayo cohort as the training cohort and Pavia cohort as the validation cohort. The CHOR model had significantly higher predictive power (*C* = 0.59) compared to the HR model (*C* = 0.56; with absolute difference in Harrell’s *C* of 0.03, 95% CI, 0.01–0.06; *p* = 0.006) as well as when compared to the OR model (*C* = 0.56, with absolute difference in Harrell’s *C* of 0.03, 95% CI: 0.01–0.05; *p* = 0.009). In the subset of patients with cardiac involvement, the CHOR model (*C* = 0.62) had significantly higher predictive value compared with the HR model (*C* = 0.57, with absolute difference in Harrell’s *C* of 0.05, 95% CI: 0.02–0.07; *p* = 0.001); but not when compared to achieving a cardiac response (*C* = 0.60, with absolute difference in Harrell’s *C* of 0.02, 95% CI: −0.01 to 0.04; *p* = 0.2). In patients with renal involvement, the CHOR model (*C* = 0.58) had significantly higher predictive value when compared with achieving a renal response (*C* = 0.52, with absolute difference in Harrell’s *C* of 0.06, 95% CI: 0.02–0.09; *p* = 0.2); however this difference was not significant when compared to the HR model (*C* = 0.56, with absolute difference of 0.02, 95% CI −0.01 to 0.05; *p* = 0.25)

## Discussion

At present, there is no available model in AL amyloidosis that allows for concurrent analysis of hematologic and organ responses (especially responses in multiple organs) in a group of patients. Our retrospective study assessed response with treatment in two independent cohorts of patients with AL amyloidosis to develop and validate a model integrating simultaneous assessment of both HR and OR. Importantly, the model was able to predict OS in both cohorts, with greater predictive power compared with HR or OR assessed in isolation. This model can be used as a surrogate end-point for rapid assessment of clinical trials. This would allow for shorter duration of follow-up and enable faster completion of these studies. This model can be incorporated in studies designed to make early treatment changes based on response. It can also be easily integrated into clinical practice for prognostication and integrating data across different therapeutics for clinical decision making.

The testing and validation cohorts were large independent cohorts from amyloidosis referral centers with long-term follow-up data. The majority of patients in both cohorts had cardiac involvement and about one-half had involvement of more than one major organ. Overall treatment patterns observed in our cohorts are similar to other cohorts reported over this time period^27,30^. Rates of transplant were strikingly different in the two cohorts (Mayo: 41%, Pavia: 1%), which suggests that the results and the CHOR model are generalizable to patients managed with different treatment approaches.

As there is no current method to evaluate multiple organ responses simultaneously, we first developed a combined parameter to assess OR. In both cohorts, patients who achieved response in all organs (AOR) had significantly better OS than those achieving response in some (MOR) or none (NOR) of the involved organs. When comparing MOR and NOR subgroups, there was no difference in OS in the overall Mayo Clinic cohort. However, there was clearly a significant difference MOR vs. NOR groups when evaluating patients with more than one organ involvement, which is the group where mixed or discordant organ responses are possible. This OR end-point was then combined with HR in a simple, easy to use CHOR model which scored patients from low to high if they achieved response vs. not. This scoring was derived from HR for survival from Cox proportional hazards analysis. Patients could be categorized into two distinct groups with different survival outcomes based on OR and HR assessment at the 6-month landmark in both cohorts. This model was able to distinguish between patients at the 12-month landmark as well. Moreover, this composite model had better predictive power for OS than either HR or OR in isolation in both the test and validation cohorts. The absolute survival outcomes in various groups were different in the Mayo Clinic and Pavia cohort. These differences are likely attributable to several factors including the differences in treatment, specifically the rates of stem cell transplant, which were strikingly different (41% vs. 1%) and possibly the responses in individual organs, particularly cardiac response, which is the major driver of survival in AL amyloidosis. Further, while the absolute survival outcomes differed in the two cohorts, the general magnitude of difference was similar.

In subset analysis of patients with cardiac involvement, the composite model had better predictive value compared with HR, but not cardiac response. This may be due to lack of adequate power or alternatively, cardiac response may be the main driving factor impacting survival. On the other hand, in the subset of patients with renal involvement, the CHOR model performed better compared to achieving renal response in predicting patient survival, but not when compared to HR. This may again be due to lack of adequate power or the fact that achieving renal response does not impact OS^27^. However, renal response remains important as it is a strong predictor for renal failure requiring dialysis as shown in our study and prior reports^27^. As over two-thirds of patients with AL amyloidosis can have involvement of more than one organ^4^, the combined CHOR model, with superior predictive value would be applicable to all patients with AL amyloidosis.

Overall, patients who achieve both OR and HR early in disease course have superior outcomes. This finding is reassuring and the development of a model which can be used systematically to assess both responses is a novel contribution of our study. The current model is able to integrate the relative improvements in hematological and organ parameters to provide a unified readout that can be used in clinical trials, as well as in clinical practice for potentially altering treatment approaches. Our study has limitations given its retrospective design, and heterogeneous nature of treatment received by patients. Moreover, the survival outcomes of patients in the two cohorts are different, likely related to baseline risk and differences in therapies used. However, development of a model in a real world scenario with an independent validation cohort results in wider applicability of the model. Previous cohorts of patients that have been used for development of HR and OR criteria for amyloidosis have also been treated in a heterogeneous manner^16,27^.

In conclusion, we have developed a model in AL amyloidosis to assess multiple organs concurrently, as well as integrate both HR and OR assessments to determine early clinical benefit with treatment, supporting its use as a surrogate end-point in clinical trials and compare outcomes with different therapeutic approaches. Future studies incorporating this endpoint should be designed to evaluate the utility of changing treatment in patients not achieving this endpoint.

## Supplementary information

Supplementary Data
