# Supplementary material for: A validated composite organ and hematologic response model for early assessment of treatment outcomes in light chain amyloidosis
Source: Blood Cancer J. 2020 Apr 14;10(4):41. doi: 10.1038/s41408-020-0306-5 (PMC7156647; doi:10.1038/s41408-020-0306-5)
Supplement: Supplementary file 1 — Supplementary Data [file 41408_2020_306_MOESM1_ESM.pdf]

Supplementary data:

Supplemental Methods:

Organ response assessment criteria: Cardiac response was defined as 30% decrease in NT-ProBNP (and at least 300 pg/mL) in patients with a baseline NT-proBNP of at least 650 pg/mL. Progression was defined as a sustained 30% and at least 300 pg/mL rise in NT-ProBNP or sustained 33% increase in troponin-T. Renal response was evaluated only in patients who were not on dialysis at diagnosis. Response was defined as 30% reduction in 24 hour urine protein in absence of $\geq$25% worsening in glomerular filtration rate. Progression was defined as $\geq$ 25% increase in GFR. Liver response was defined as 50% reduction in alkaline phosphatase, while progression was as 50% increase.

Supplemental Results:

In patients with involvement of both heart and kidney and who achieved cardiac response by six months, status of renal response did not impact survival further. In the Mayo group, median OS for patients with renal response (n=17) vs. not (n=16) was not reached in both groups, p=0.2 (estimated 5-year OS: 86% vs. 58%). Similarly, in the Pavia group median OS for patients with renal response (n=36) vs. not (n=30) was 100 months vs. not reached, p=0.8. On the other hand, in patients not achieving a cardiac response at six months, median OS in the Mayo group for those achieving a renal response (n=38) vs. not (n=66) was 67 vs. 50 months, p=0.2. In the Pavia cohort, median OS for those who achieving renal response (n=56) vs. not (n=108) was 50 vs. 31 months, p=0.83

Figure S1:

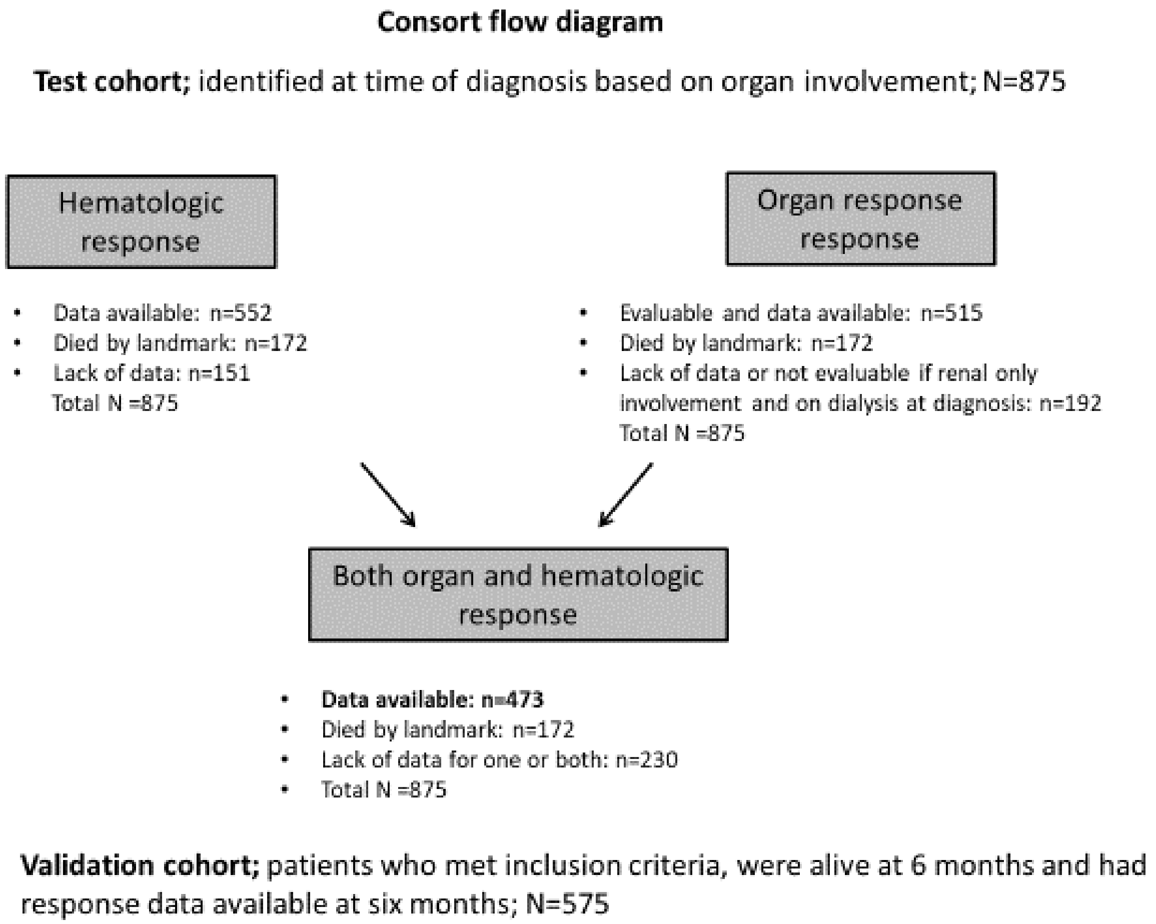
Consort flow diagram
Test cohort; identified at time of diagnosis based on organ involvement; N=875
Hematologic response
Organ response response
Data available: n=552
Died by landmark: n=172
Lack of data: n=151
Total N =875
Evaluable and data available: n=515
Died by landmark: n=172
Lack of data or not evaluable if renal only involvement and on dialysis at diagnosis: n=192
Total N =875
Both organ and hematologic response
Data available: n=473
Died by landmark: n=172
Lack of data for one or both: n=230
Total N =875
Validation cohort; patients who met inclusion criteria, were alive at 6 months and had response data available at six months; N=575

Figure S2: Combined organ response based on stratification by Mayo 2012 stage in patients with > 1 organ involvement

Organ response at 6 months, Mayo (>1 organ involved):

Mayo stage 1 and 2 | Mayo stage 3 and 4

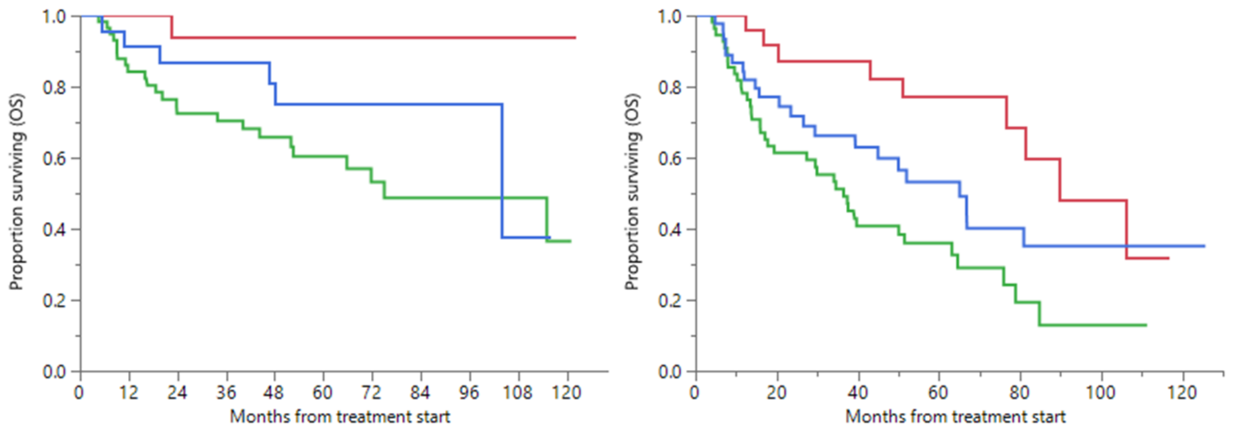

AOR, N=16, not reached
MOR, N=23, 104 months
NOR, N=58, 75 months
P=0.01

AOR, N=27, 90 months
MOR, N=55, 65 months
NOR, N=55, 37 months
P=0.002

AOR (All organ response)
MOR (Mixed organ response)
NOR (No organ response)

AOR: All organ response, MOR: Mixed organ response, NOR: No organ response

Figure S3: Overall survival in patients with cardiac involvement based on achieving all organ response (AOR), mixed organ response (MOR) and no organ response (NOR) at six months from start of first-line therapy (a) Mayo Clinic (b) Pavia cohort

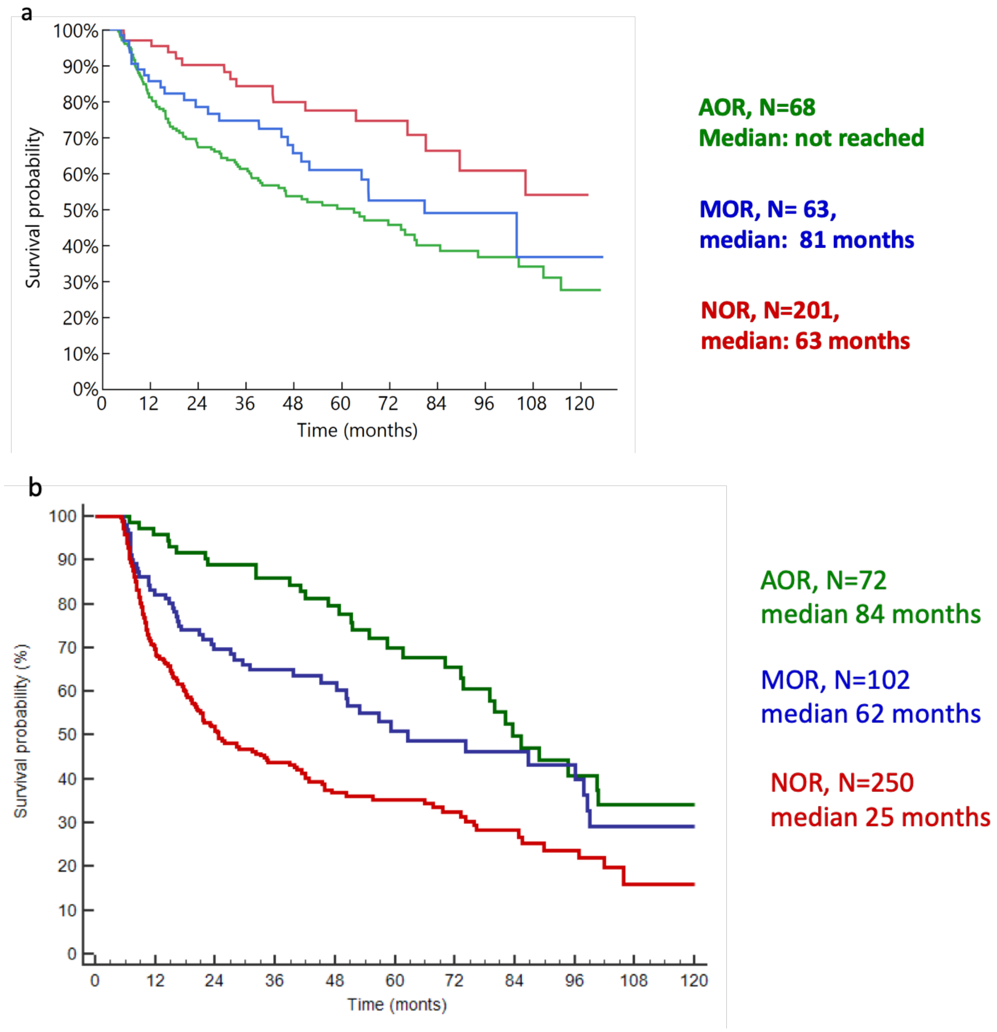

Figure S4: OS by CHOR groups in sub-groups of patients with Mayo 2012 stage 1-4 (6 month time-point)

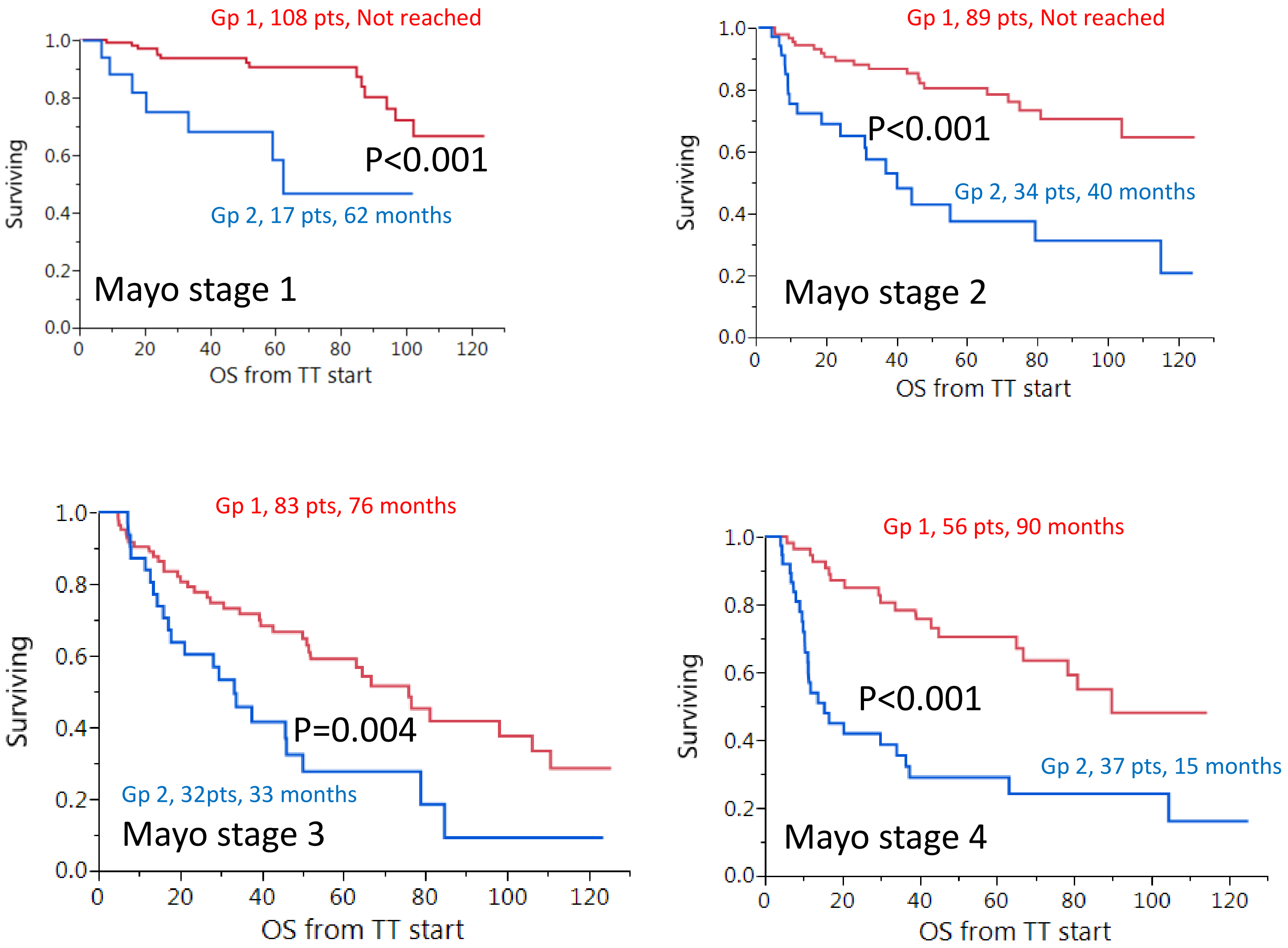

Figure S5: CHOR groups based on 12 month response (Mayo Cohort)

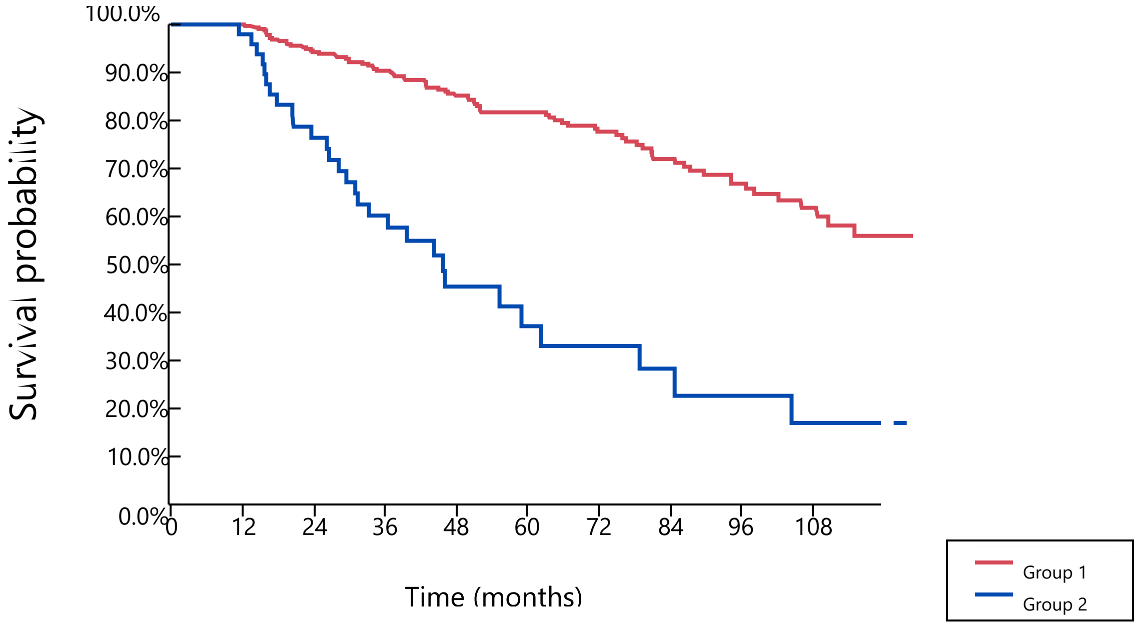

Group 1, N=335, median OS, not reached (111-not reached)

Group 2, N=50, median OS: 46 months (31-62)
